# A Case Report of Nearly Missed Renal Tubular Acidosis in the Setting of Sjögren’s Syndrome

**DOI:** 10.7759/cureus.34899

**Published:** 2023-02-12

**Authors:** Rachael Caretti, Christopher Fiechter, Natan Babek, Travis Smith, Happy Sadiek

**Affiliations:** 1 Medicine, Lake Erie College of Osteopathic Medicine, Bradenton, USA; 2 Internal Medicine, Lake Erie College of Osteopathic Medicine, Bradenton, USA; 3 Clinical Curriculum Integration & Assessment, Lake Erie College of Osteopathic Medicine, Bradenton, USA; 4 Nephrology, Lake Erie College of Osteopathic Medicine, Bradenton, USA

**Keywords:** sjogren syndrome, distal renal tubular acidosis, acute kidney injury, chronic kidney diseases, non-anion gap metabolic acidosis

## Abstract

The association of renal tubular acidosis (RTA) and Sjögren’s syndrome (SS) has been well-documented in the literature previously but is often undiagnosed in clinical practice. In this case report, we present a case of a woman with distal RTA who presented with nausea, vomiting, and confusion. The case shows the diagnostic value of urine studies when evaluating a patient who has exaggerated and unexplained electrolyte losses and how this will change management. Recognizing the extra glandular manifestations of patients with SS is important for patient care to prevent delays in care and treatment.

## Introduction

Pathophysiology of Sjögren’s syndrome

Primary SS is an autoimmune condition that more commonly affects females and often presents with xerostomia, xerophthalmia, fatigue, and/or arthralgias [1]. It is characterized by immune-mediated inflammation and destruction of exocrine glands [2]. Its diagnosis is based on the International Consensus Criteria for Sjögren’s Syndrome, which requires that a patient has four of six diagnostic criteria; one of which must be a positive salivary gland body or antibodies to Sjögren's-syndrome-related antigen A or Sjögren's-syndrome-related antigen B. Other diagnostic criteria include subjective ocular symptoms, subjective oral symptoms, objective eye dryness (example: positive Schirmer test), or objective salivary gland involvement (example: positive salivary gland scintigraphy) [3]. Systemic involvement is a common feature of SS and may affect the lungs, gastrointestinal tract, skin, and kidneys [1]. Renal manifestations often go unnoticed until there is an acute event such as hypokalemic paralysis, renal calculi, or osteomalacia [3]. Chronically, it may also present as interstitial nephritis, systemic acidosis, or progressive chronic kidney disease [1]. Interstitial nephritis is the most common renal manifestation of SS and glomerulonephritis occurs less often as a result of cryoglobulinemia. Diagnostic workup of renal disease in SS is important to guide management; in some cases, renal biopsy may be necessary. Rituximab is one of the current therapies offered to patients with SS and renal involvement given the suspected role of B-cell activation [1]. Distal renal tubular acidosis (RTA) has been reported in the setting of primary SS but causation has not been well-established. It has been hypothesized that there may be deficient H+-ATPase pump function related to immune-mediated cellular apoptosis [4]. Another study suggests that RTA may be mediated by anti-carbonic anhydrase antibodies [5].

Pathophysiology of distal renal tubular acidosis

Distal RTA is caused by impaired distal acidification of the urine [6]. In normal renal physiology, alpha-intercalated and principal cells in the collecting tubule secrete protons [7]. Sodium is reabsorbed through the epithelial Na+ channel on the apical membrane of principal cells. This causes an electronegative potential in the tubular lumen that leads to the secretion of protons. Alpha-intercalated cells then express the H+-ATPase enzyme leading to proton excretion. Carbonic anhydrase II plays a role in the production of protons and bicarbonate ions within alpha-intercalated cells. Excess H+ ions are also excreted into the tubular lumen via the H+/K+-ATPase, but this exchange occurs to a lesser degree [7]. Distal RTA occurs when H+ is not secreted into the urine, resulting in a urine pH above 5.5 in the setting of systemic acidosis. The exact mechanism of distal RTA in SS has not been well-established but studies have shown an absent H+-ATPase enzyme in some patients [6]. Additionally, other studies report antibodies against carbonic anhydrase II. The management of distal RTA in patients with autoimmune diseases has not been well-studied and primarily focuses on normalizing the acid-base balance [7]. Alkali therapy is beneficial in reducing nephrolithiasis formation but in the cases of delayed diagnosis end-stage renal disease may be unavoidable [6].

## Case presentation

A 72-year-old female presented to the emergency department (ED) via emergency medical services after developing two weeks of fatigue, poor appetite, nausea, vomiting, and confusion. She had associated nausea and vomiting. Her husband was concerned about possible aspiration and noted that she had been spitting up when drinking sips of water. Additionally, she had left-sided parotid gland swelling, which developed about five days prior to admission, and was treated with clindamycin and amoxicillin clavulanic acid by her PCP without resolution. Her past medical history was significant for SS, chronic kidney disease (CKD) with unknown baseline creatinine, nephrolithiasis, hypothyroidism, gastroesophageal reflux disease, restless leg syndrome, herpes simplex virus, and a remote history of breast cancer and aspergillosis. Of note, within the past year, the patient had two prior stays in the intensive care unit (ICU) at another facility, with sepsis secondary to pneumonia, but those records were unobtainable. Her home medications included gabapentin, hydroxychloroquine, levothyroxine, omeprazole, prednisone, and valacyclovir.

Initial vitals were a blood pressure of 96/53 mmHg, a heart rate of 116 beats per minute (bpm), a respiratory rate of 16/minute, pulse oximetry of 97% on room air, and an oral temperature of 38 degrees Celsius. On exam, the patient appeared to be in mild distress and was somnolent. She was oriented to person and place but not time or situation. She had dry oral mucosa and mild left-sided facial swelling. She was without murmurs and had normal peripheral perfusion. Her lungs were clear to auscultation and a neurologic exam revealed normal motor, speech, coordination, and sensory functions but showed she was slow to respond.

**Figure 1:**
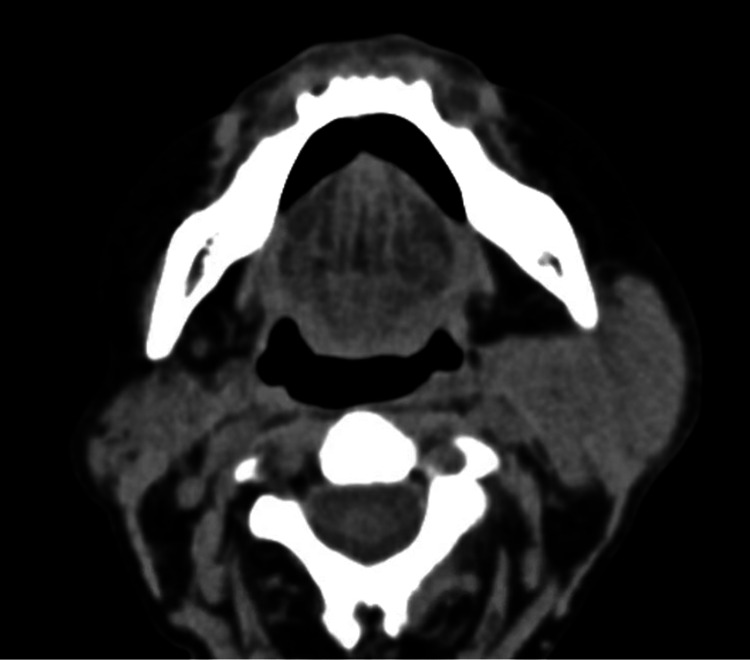
CT scan showing left parotid enlargement

Neurologic workup included a head/maxillary/facial bone computed tomography (CT) scan, which showed no acute intracranial findings and asymmetric enlargement of the left parotid gland with recommendations to correlate clinically for parotitis (Figure 1).

**Figure 2:**
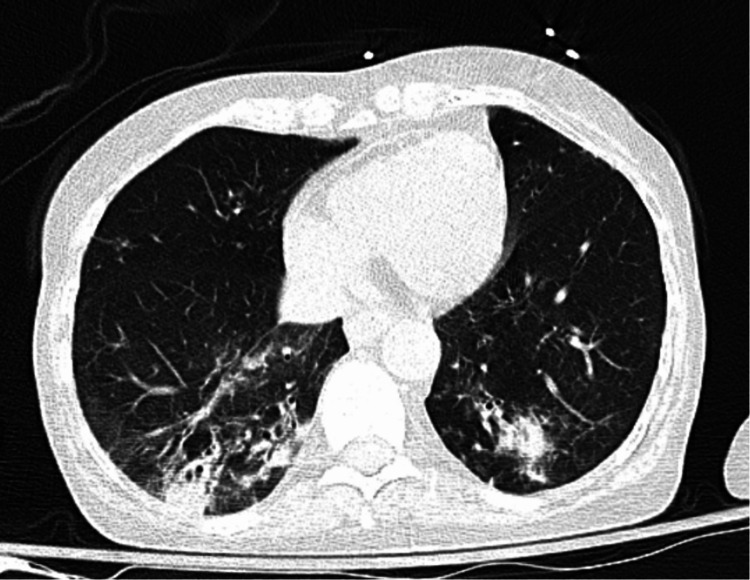
CT scan of the chest with bilateral airspace disease

She had an arterial blood gas that revealed a critically low pH at 7.14 and partial pressure of carbon dioxide (PCO2) was also low at 17.4 mmHg. Her partial pressure of oxygen (PO2) was 113 mmHg and bicarbonate (HCO3) was low, at 6.1 mmol/L, with a base deficit of -23.0 mmol/L. CT of the chest showed patchy airspace disease in the bilateral lower lobes and right middle lobe suggestive of multifocal infection as well as pulmonary nodules in the right upper lobe (Figure 2). She was negative for coronavirus disease 2019 (COVID-19) infection.

Electrocardiogram performed in the ED showed sinus tachycardia at 114 bpm with no ST-T changes and normal PR and QRS intervals. Troponin was within normal limits.

Urinalysis was positive for protein, trace ketones, and moderate blood. Leukocyte esterase was negative but there were six white blood cells (WBC) per high-powered field and rare bacteria. Significant results from her chemistry panel included a blood urea nitrogen (BUN) of 42 mg/dL, creatinine of 2.4, hyperchloremia at 125 mmol/L, carbon dioxide of 8 mmol/L, and an anion gap of 11 mmol/L; the rest of her results can be found in Table 1. Her CBC showed a WBC that was elevated at 31.20 k/mcL with 95% neutrophils and no bands. Hemoglobin and hematocrit were low at 11.3 g/dL and 35.5% with thrombocytosis at 555 k/mcL. Procalcitonin was elevated at 10.11 ng/mL. Protime and international normalized ratio (INR) were both within normal limits. Lactic acid was within normal limits at 1.2 mg/dL.

**Table 1:** Results from the chemistry panel plus added-on chemistry labs BUN: blood urea nitrogen; eGFR: estimated glomerular filtration rate

Lab	Result	Units
BUN	42	mg/dL
Creatinine	2.4	mg/dL
eGFR	21	mL/min/1.73m2
Chlroide	125	mmol/L
Calcium	10.7	mg/dL
Sodium	144	mmol/L
Potassium	3.7	mmol/L
Magnesium	2.1	mg/dL
Phosphorous	3.6	mg/dL
Glucose	82	mg/dL
Carbon Dioxide	8	mmol/L
Anion Gap	11	mmol/L

In the ED the patient was given a liter of lactated ringers fluid bolus and was started on cefepime and vancomycin for presumed sepsis. Plans for admission with an initial diagnosis of metabolic encephalopathy, multifocal pneumonia, sepsis, parotitis, metabolic acidosis, and acute-on-chronic renal insufficiency.

The patient was evaluated by the hospitalist service and evaluated for PCU admission. Her current antibiotic regimen and intravenous fluids (IVF) were continued. Due to her non-anion gap metabolic acidosis, a bicarbonate drip was initiated, and nephrology was consulted for further recommendations. Her home prednisone, 5 mg, was administered to decrease the risk of adrenal insufficiency. The intensivist was also consulted for possible ICU admission given the severity of her acidosis.

The intensivist evaluated the patient and believed it would be beneficial for her to be stabilized in the ICU. The intensivist also assessed the patient and believed that her sepsis was likely due to pneumonia and parotitis. Her non-anion gap metabolic acidosis was attributed to poor oral intake and dehydration in the setting of emesis. Her thyroid-stimulating hormone (TSH) and free thyroxine levels were added on and within normal limits. A CT abdomen and pelvis (CTAP) was added on to rule out other causes of sepsis.

During afternoon rounds, the patient had not yet produced any urine, and a bladder scan showed she had one liter of urine in her bladder. A foley catheter was placed for acute urinary retention. CTAP showed ascending colitis and given her recent treatment with clindamycin, the patient was started on oral vancomycin for possible Clostridioides difficile (C. difficile). A nasogastric tube was also placed for tube feeds given the patient’s poor baseline nutritional status. Additionally, after the speech pathologist's examination, it was felt that she had severe dysphagia leading to aspiration and recurrent pneumonia. She received a stress dose of dexamethasone, and her home dose of prednisone was continued. The current antibiotic regimen and bicarbonate drip were also continued at this time.

**Figure 3:**
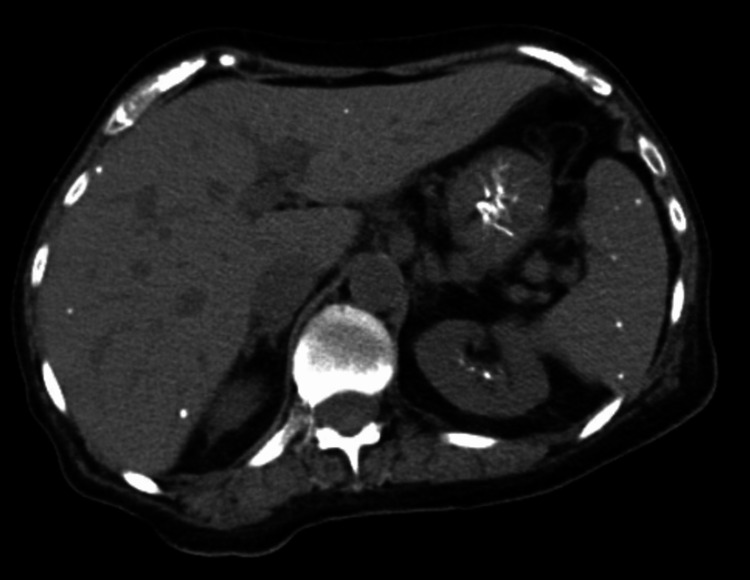
CT of the abdomen showing hepatic and splenic calcified granulomas, as well as nephrolithiasis

Later that day, a repeat CTAP was ordered given the patient’s down-trending hemoglobin to rule out a retroperitoneal bleed. There was no evidence of retroperitoneal hematoma, improving colitis, multiple bilateral nephrolithiasis, and multiple calcified hepatic and splenic granulomas (Figure 3).

Nephrology was concerned about an RTA, given she had a hyperchloremic normal anion gap metabolic acidosis in the setting of SS. Urine electrolytes were ordered and nephrology recommended electrolyte repletion and continuation of the bicarbonate drip while results were pending. 

She had no acute events overnight and when evaluated on hospital day three, she was becoming more interactive and talking compared with admission. Her serum bicarbonate normalized from admission. At this point, the bicarbonate drip was discontinued, and the patient was determined to be clinically stable and downgraded to the PCU.

**Figure 4:**
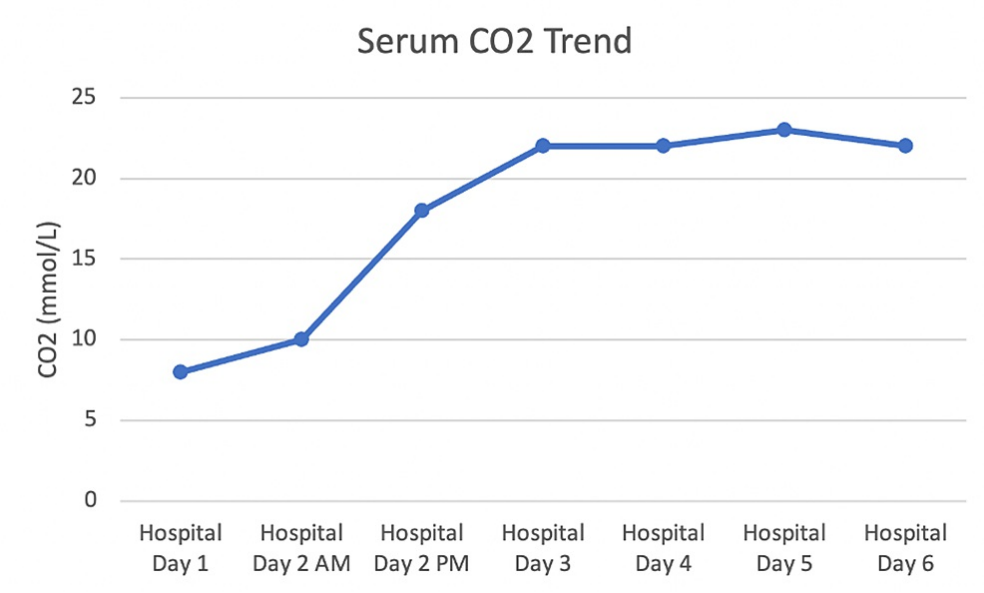
Trend of the patient’s CO2 levels throughout the admission

Her urinary electrolytes returned and revealed a positive urine anion gap at 15.0 mEq/L (Table 2), clinically consistent with a distal RTA with recommendations to start oral sodium bicarbonate therapy, which was to be continued outpatient as well (Figure 4).

**Table 2:** Urine electrolyte studies

Urine Electrolytes	
Creatinine (mg/dL)	25.5
Sodium (mEq/L)	75
Potassium (mEq/L)	16
Chloride (mEq/L)	76

**Figure 5:**
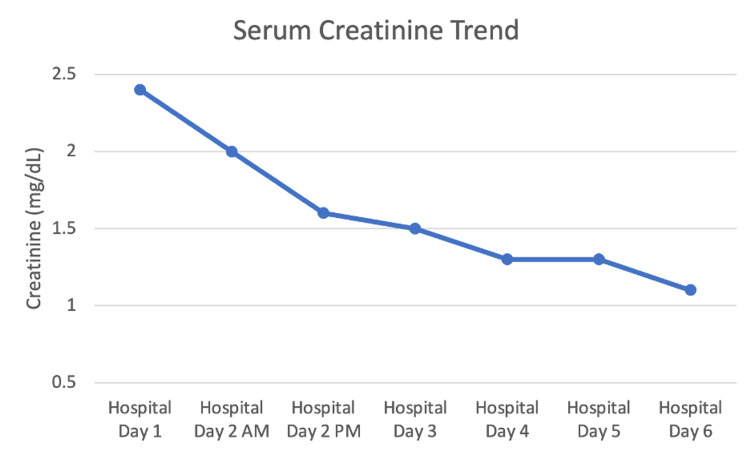
Trend of patient’s serum creatinine throughout her hospitalization

Following her transfer, she was now alert and oriented, resting comfortably on room air. Her antibiotics were downgraded to ceftriaxone and metronidazole as her cultures as well as her methicillin-resistant Staph aureus polymerase chain reaction (PCR), legionella, and urinary Streptococcus antigens returned negative. Stool samples for C. difficile were negative so oral vancomycin was discontinued. She did have positive immunoglobulin M (IgM) and immunoglobulin G (IgG) titers for Mycoplasma pneumoniae, so the recommendation was to add azithromycin to her antibiotic regimen. Her creatinine continued to improve (Figure 5) with in vitro fertilization (IVF) so her foley catheter was removed; she was producing adequate urine without retention.

**Figure 6:**
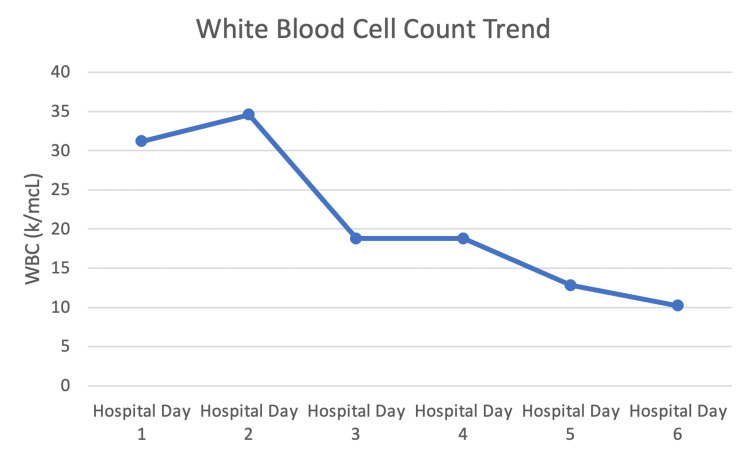
Trend of patient’s WBC over the course of the hospital stay

By hospital day five continued to do well with a down-trending WBC (Figure 6). She developed mild hypernatremia, so her IVF was switched to ½ normal saline. Her hemoglobin continued to decrease to 6.5 g/dL, so one unit of packed red blood cells (pRBCs) was transfused.

Nephrology continued to follow the patient and said that her hypocalcemia in the setting of hypoalbuminemia could be followed outpatient and there was nothing to do acutely. Her serum creatinine continued to downtrend, and her acidosis resolved with bicarbonate therapy.

The patient had no acute events overnight into hospital day six. Her WBC down trended to within normal limits and antibiotics were switched to the oral regimen. After the transfusion of one unit pRBCs, her hemoglobin was stable at 8.3 g/dL. Her renal function also improved to her reported baseline. At this point, she was clinically stable for discharge with outpatient follow-up.

## Discussion

This is an interesting case of a woman with probable chronic distal RTA that went undiagnosed for years prior to her admission. Early recognition of RTA can lead to clinically significant outcomes due to targeted treatment, and thus should always be considered a differential diagnosis when at-risk patients present in an emergency setting. In this patient’s case, diagnostic evidence supporting distal RTA included hyperchloremic metabolic acidosis with a normal anion gap, an arterial pH of 7.14, a urine pH greater than 5.5, and a positive urine anion gap [6]. It is suspected that SS was the primary contributor to the development of RTA for this patient because of the temporality of parotid gland swelling unresolved by antibiotics. Specifically, it is suspected that the etiology of this patient’s parotitis was Sjögren’s autoimmune infiltration, triggered by an active pulmonary infection [8]. If profound enough, this autoimmune exacerbation could compromise carbonic anhydrase II activity and thus provoke distal RTA [7].

However, other potential causes of this patient’s RTA cannot be discounted. For example, the patient presented with obstructive uropathy of undetermined etiology, which may have predisposed the patient to RTA due to urinary stasis [9]. Additionally, volume depletion can lead to impaired distal acidification of the urine, and given her presentation with dehydration, it is important to consider this as a cause of her RTA. In the setting of multiple nephrolithiasis and SS and urinary sodium of 75, it is less likely to be the etiology in this case. Other autoimmune conditions, such as Hashimoto’s thyroiditis, can also underlie RTA, although this patient’s thyroid function was within normal limits and her history of hypothyroidism was not further investigated. It is possible other medications were contributing but undisclosed such as non-steroidal anti-inflammatories or other over-the-counter medications. It should be considered in subsequent literature the degree to which multiple risk factors may compound to increase one’s predisposition for RTA. Regardless, this case highlights the importance for clinicians to recognize the many potential causes of RTA because identifying the primary contributors will expedite effective treatment. Given that the above patient's history and laboratory evaluations were clinically consistent with RTA, the decision was made that a kidney biopsy was not necessary. In the appropriate clinical setting where a cause of renal dysfunction is not readily identified, a biopsy could be beneficial to further investigate disease etiology.

Other differential diagnoses were considered as well, given this patient’s presentation. Interstitial nephritis was considered a possibility in the context of her history of SS and recent amoxicillin use, however, this patient did not have the relevant clinical findings such as rash or high fever. It was also considered that this patient might have hypercalcemia of sarcoidosis given the extensive hepatic and splenic calcified granulomas. However, the absence of pulmonary involvement is atypical for sarcoidosis, and thus this was considered less likely given the CT findings and prior history of aspergillosis [10].

This patient was treated initially and predominantly on the premise of acute kidney injury (AKI) on CKD alone, delaying the administration of sodium bicarbonate until a blood gas was obtained the following day. Thereafter, sodium bicarbonate was discontinued due to improved blood CO2 levels but subsequent urine electrolytes revealed an unresolved urine anion gap. This case supports increased utilization of RTA diagnostics, such as urine electrolytes, urine pH, and evidence of nephrolithiasis, to identify the underlying cause and, if relevant, determine the timing of sodium bicarbonate administration and discontinuation. Additionally, this case demonstrates that recognizing clinical manifestations of autoimmune flare-ups, such as SS, presents utility in RTA diagnostics. This patient’s parotitis was presumed infectious and thus was medically treated with antibiotics. Consideration for autoimmune infiltration, alternatively, as the pathophysiology of her parotitis, may have advanced the recognition and treatment of RTA secondary to SS in this case.

## Conclusions

This case illustrates the insidious nature that distal RTA may have when presenting with SS. Early detection is crucial, as prompt management can prevent further complications and renal decline. Evidence of hyperchloremic normal anion gap metabolic acidosis provided the initial tip to the diagnosis and could be a marker for future investigations. The importance of urinary studies should be emphasized and used as a tool to distinguish between different causes of electrolyte disturbances. With appropriate management and resolution of her infections, the patient’s acidosis resolved and her creatinine and renal function improved.
